# Saudi Secondary Prevention Survey Study in Patients with Prior Acute Myocardial Infarction (4S Registry): Study Design and Pilot Phase Results

**DOI:** 10.3390/jcdd13020100

**Published:** 2026-02-20

**Authors:** Rakan K. Alhabib, Naji Kholaif, Mohammed Mahmoud, Abdulrahman Alnwiji, Fayez Al Zubair, Hassan Almir, Ahmad Saad Alzoman, Abdulmalik Abdullah Alqahtani, Rasha Alsharkawy, Zahra Mohammed Albahar, Khalid F. Alhabib

**Affiliations:** 1 College of Medicine, Alfaisal University, Riyadh 50927, Saudi Arabia; 2 Heart Centre, King Faisal Specialist Hospital & Research Center, Riyadh 12713, Saudi Arabia; 3 Heart Health Center, King Saud Medical City, Riyadh First Health Cluster, Riyadh 12746, Saudi Arabia; 4 Department of Cardiac Sciences, King Fahad Cardiac Center, College of Medicine, King Saud University Medical City, King Saud University, Riyadh 11421, Saudi Arabia876ani@gmail.com (A.A.A.);; 5 King Salman Hospital, Riyadh 12769, Saudi Arabia; 6 Qatif Central Hospital, Eastern Health Cluster Ministry of Health, Qatif 32253, Saudi Arabia

**Keywords:** secondary prevention, preventative care, acute myocardial infarction, cardiovascular diseases

## Abstract

Patients with prior acute myocardial infarction (AMI) generally show low rates of achieving secondary prevention targets. Here we evaluated adherence to guideline-recommended secondary prevention strategies after AMI in Saudi Arabia. This ambispective multicenter cohort study included consecutive patients seen for follow-up visits 6–24 months after hospitalization for AMI. A standardized questionnaire was used to evaluate control of blood pressure (<130/80 mmHg), HbA1c (<7%), LDL-C (<1.4 mmol/L), lipoprotein(a) (<50 mg/dL), body mass index (18.5–24.9 kg/m^2^), physical activity targets, smoking habits, guideline-directed medical therapy (GDMT), and referral to cardiac rehabilitation. Among 108 AMI patients (mean age 58.4 ± 10.9 years; 80.6% male; 76.9% Saudi nationals), 53.7% had uncontrolled blood pressure, ~40% uncontrolled glucose, and 67% above-target LDL-C levels. Most participants were overweight (40.7%) or obese (37%), and 28.7% achieved the physical activity targets. One-third of patients were not receiving all GDMT, 15.7% were current smokers, and 25% had been referred to cardiac rehabilitation. No patient met all guideline-recommended secondary prevention targets. This pilot study highlights gaps in secondary prevention among AMI survivors. Upcoming study phases will aim for national representation and help identify key clinical and demographic drivers to improve secondary prevention efforts across Saudi Arabia.

## 1. Introduction

Patients with known cardiovascular disease (CVD) carry an increased risk of experiencing another CVD [1,2]. However, worldwide, patients with prior acute myocardial infarction (AMI) show low rates of achieving secondary prevention targets—with only 20–40% meeting individual targets [1]. Notably, Saudi Arabia carries a high age-standardized burden of cardiovascular disease [3]. The Saudi Acute Myocardial Infarction Registry (STARS) includes 2690 AMI patients, and reveals a younger age of AMI presentation (~57 years) compared to registries in developed countries (i.e., North America and Europe) [4,5]. Patients in STARS show a high prevalence of coronary risk factors—with 58% showing hypertension, 58% having diabetes, 43% being current or past smokers, and a mean body mass index (BMI) of 28.5 kg/m^2^ [4,5,6]. Such observations are aligned with various previous studies conducted within the Saudi population and the Middle East and North Africa region [4,5,6,7]. It is presently unclear whether secondary prevention targets are being implemented in a timely manner during follow-up among patients with prior AMI events in Saudi Arabia. In the present work, we have described the overall study design of the Saudi Secondary prevention Survey Study in AMI (4S Registry), investigated the adherence to guideline-recommended secondary prevention strategies, and evaluated the control of key risk factors in patients with prior AMI in Saudi Arabia.

## 2. Materials and Methods

### 2.1. Study Design and Setting

This ambispective (retrospective–prospective) proof-of-concept study (pilot phase) was conducted among five hospitals (three tertiary and two non-tertiary) in Saudi Arabia. Tertiary hospitals were defined as those equipped with a cardiac care or intensive care unit (CCU/ICU), a cardiac catheterization laboratory, and cardiac surgery services. Non-tertiary hospitals were defined as those not equipped with any of the aforementioned services. We retrospectively identified consecutive follow-up patients with a prior hospitalization for a first or recurrent AMI, including non-ST-elevation MI (NSTEMI) and ST-elevation MI (STEMI). AMIs were defined following the 2023 ESC guidelines [8]. Eligible patients were males and females, who were ≥18 years of age at the time of inclusion, and whose index MI event occurred 6–24 months prior to enrolment. All necessary data were collected once during the allocated timeframe. Patients who did not meet these criteria were excluded.

The following variables were prospectively obtained from the medical records: blood pressure, glucose levels, LDL-C levels, lipoprotein(a), height, weight, physical activity, smoking habit, prescription of the four pillars of guideline-directed medical therapy (GDMT), and access to cardiac rehabilitation. Included laboratory results had to correspond to the clinic visit date, or have been obtained within one month of the inclusion date. Lab results from outside of this timeframe were not analyzed, and the corresponding variables were marked as “unmeasured” in the case report form (CRF). Data regarding smoking habits, physical activity habits, and cardiac rehabilitation referral outcomes were obtained directly from patients, either via telephone interview or during the clinic follow-up. All participants gave verbal consent prior to data collection.

### 2.2. Study Phases

A. Pilot Phase (the current study): The pilot results are reported in the present study, which was designed to assess the validity and feasibility of completing the CRF in real-world clinical practice, and to identify potential logistic challenges.
B. Phase I: This phase will involve 6–8 hospitals, with a planned total sample size of 600 (~100 patients per hospital). The aim will be to provide an initial overview of adherence to guideline-recommended secondary prevention targets, and to compare key outcomes across various demographics (e.g., tertiary vs. non-tertiary hospitals, male vs. female patients).
C. Phase II: This phase will establish a nationwide registry designed to provide a comprehensive representation from all regions of Saudi Arabia.

### 2.3. Objectives

The primary objective is to determine the adherence to guideline-recommended secondary prevention strategies during follow-up after AMI. Secondary objectives are to compare these findings between tertiary versus non-tertiary hospitals, between males versus females, between patients with a single versus multiple AMI events, and between patients based on the duration of follow-up relative to the event.

### 2.4. Data Collection and Management

Data collection and submission was performed once for each patient, only at the inclusion date, by senior medical students, interns, nurses, research assistants, or physicians. Investigators were required to review files, and to record and collect data. Recorded data were submitted using a standardized online case report form. Demographic and personal information were obtained from the patients’ medical records. Data collectors were required to contact the patients to obtain information regarding smoking history, cardiac rehabilitation, and physical activity. Subsequently, they would measure and record cardiovascular risk factors, as follows:

(1) *Blood pressure* measurements were recorded once during follow-up. Elevated blood pressure readings were defined as a systolic blood pressure of ≥130 mmHg and/or diastolic blood pressure of ≥80 mmHg [9].
(2) *Blood glucose* measurements were considered abnormal if HbA1c was ≥7.0% [10].
(3) *LDL-C* normal targets were set at ≤55 mg/dL (1.4 mmol/L), according to the most recent 2025 focused update of the 2019 ESC/EAS guidelines for the management of dyslipidemias [11,12].
(4) *Lipoprotein(a)* normal targets were set at <50 mg/dL (<105 nmol/L) [12,13].
(5) *Height and weight* were measured at the follow-up visit, with patients wearing light indoor clothes without shoes. BMI was calculated and <18.5 kg/m^2^ was considered underweight, 18.5–24.9 kg/m^2^ normal weight, 25–30 kg/m^2^ overweight, and ≥30 kg/m^2^ obese [14].
(6) *Physical activity* was self-reported. Patients described their exercise habits, if any, and the type(s) of physical activity performed. Three questions were included, in accordance with the World Health Organization’s (WHO) weekly physical activity recommendations [15,16]. The WHO recommends that adults should do at least 150–300 min of moderate-intensity aerobic physical activity, or at least 75–150 min of vigorous-intensity aerobic physical activity; or an equivalent combination of both throughout the week [16,17]. A question about the type of physical activity was also included.
(7) *Prescription of all four pillars of GDMT*: GDMT includes angiotensin-converting enzymes inhibitors (ACE inhibitors) or angiotensin receptor blockers (ARBs), antiplatelet therapy (e.g., aspirin or other antiplatelet agents), lipid-lowering therapy (e.g., statins, ezetimibe, PCSK-9i, and inclisiran), and beta-blockers [9].
(8) *Smoking habits* were self-reported. Patients were asked three questions to address smoking habits (current, past, or non-smoker), type of smoking (cigarettes, sheesha, vape, or other), and years of smoking. Past smokers were defined as patients with a smoking cessation duration of ≥1 month.
(9) *Cardiac rehabilitation* was self-reported. Patients were asked a single question about whether they had been referred to cardiac rehabilitation.

### 2.5. Study Coordination

The overall study was coordinated, and data were monitored for completeness and correctness, by Dr. Rakan K Alhabib (Principal Investigator) and Dr. Naji Kholaif (Co-Principal Investigator).

### 2.6. Statistical Considerations

All statistical analyses were performed using IBM SPSS Statistics version 27. Descriptive statistics were applied to summarize socio-demographic and clinical characteristics. Continuous variables (e.g., age, blood pressure, LDL-C, glucose levels, height, weight, BMI, and time from ACS event to study inclusion) were reported as means and standard deviations, and categorical variables (e.g., BMI categories, smoking status, physical activity levels, medication use, and adherence outcomes) as frequencies and percentages. Independent sample *t*-tests were used to compare continuous variables, such as the difference in time between the ACS event and inclusion across care types. Chi-square (χ^2^) tests were employed to examine associations between tertiary versus non-tertiary care settings, and categorical indicators, including clinical control measures, lifestyle behaviors, medication use, and composite adherence variables. Composite adherence metrics were computed according to guideline-defined criteria involving pharmacological, clinical, and lifestyle components. A significance level of *p* < 0.05 was used to determine statistical significance across analyses.

### 2.7. Ethical Considerations

All data were retained by the authors, and used only for research purposes. Data were electronically stored in an encrypted file to ensure the security and privacy of information. This study was approval by the Institutional Review Board (IRB) of King Faisal Specialist Hospital (RAC#: 2251087) on 14 April 2025. The need for written consent was waived because *The 4S Registry* is an observational registry that included the collection of routine clinical data about patients during their post-AMI follow-up, who were managed by their own physicians, following the standard usual care and without any extra testing or interventions. Verbal consent was obtained upon contacting the patients, and was recorded on their corresponding medical files.

## 3. Results

Between July to September 2025, we enrolled 108 participants, with a mean age of 58.4 years (SD, 10.9 years). The majority were male (80.6%) and Saudi nationals (76.9%). In terms of the level of care, 75% of the participants were recruited from tertiary care centers, while 25% received care at non-tertiary hospitals. The mean time of inclusion was 1.29 ± 0.51 years (Table 1).

The majority (79.6%) of patients had experienced a single AMI event, while 20.4% reported multiple ACS events. The mean systolic blood pressure was 127.7 mmHg (SD, 17.8 mmHg), and the mean diastolic blood pressure was 73.9 mmHg (SD, 11.7 mmHg). Regarding biochemical parameters, the mean low-density lipoprotein cholesterol (LDL-C) level was 1.92 mmol/L (SD, 0.92 mmol/L), and the average glycated hemoglobin (HbA1c) was 7.09% (SD, 1.81%). The mean height and weight were 166.1 cm (SD, 7.2 cm) and 78.3 kg (SD, 15.8 kg), respectively, yielding a mean body mass index (BMI) of 28.3 kg/m^2^ (SD, 5.1 kg/m^2^). In terms of BMI categories, 40.7% of participants were overweight, 37.0% were obese, 20.4% had a normal weight, and 1.9% were underweight (Table 1).

**Figure 1:**
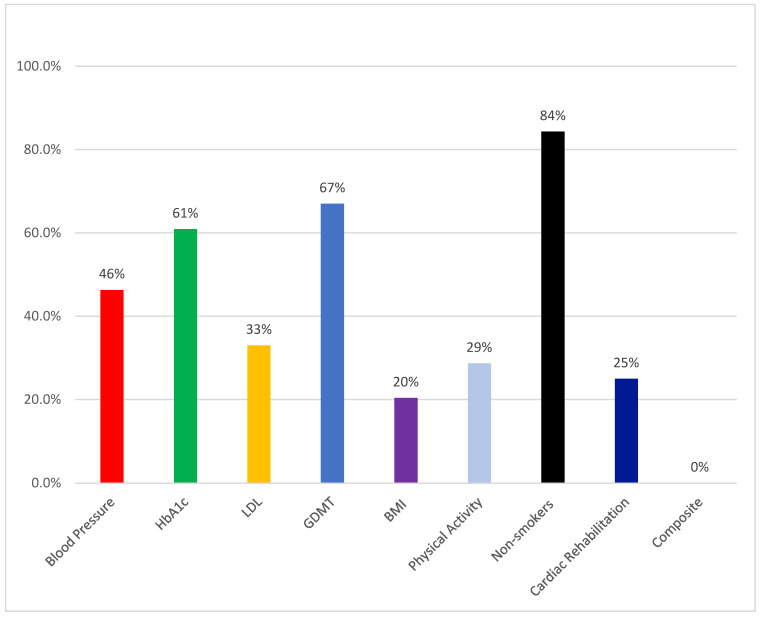
Achievement of secondary prevention targets.

Figure 1 summarizes the distribution of controlled clinical and behavioral variables according to hospital type. With regard to blood pressure control, target values (<130/80 mm/Hg) were achieved by 44.4% of patients in tertiary hospitals, and 51.9% in non-tertiary hospitals. This difference was not statistically significant: χ^2^(1, *n* = 108) = 0.44, *p* = 0.506 (see ESM 1).

Among patients with available HbA1c data (*n* = 87), measures of glycemic control indicated that 60.9% achieved the recommended target of HbA1c < 7%. Glucose control was achieved by 55.9% of tertiary-care patients and 78.9% of non-tertiary patients, with no significant difference between these groups: χ^2^(1, *n* = 87) = 3.28, *p* = 0.07. With regard to lipid profile control, 67.0% had uncontrolled LDL-C levels, while the goal (LDL-C < 1.4 mmol/L) was achieved by 36.9% of tertiary-care patients, and 21.7% of non-tertiary participants: χ^2^(1, *n* = 88) = 1.75, *p* = 0.186. Lipoprotein(a) was measured in only one participant (0.9%) (see Tables S1 and S2).

Regarding BMI, 20.4% achieved normal BMI levels, and the distribution did not significantly differ across hospital types: χ^2^(1, *n* = 108) = 3.27, *p* = 0.352. Most participants were overweight (40.7%) or obese (37.0%). The rate of adherence to all four GDMT medications was 67% among all patients, and was 63% among tertiary-care patients versus 77.8% among non-tertiary patients: χ^2^(1, *n* = 108) = 1.98, *p* = 0.159. ACE inhibitors or ARBs were used by 75.9% of patients. Patients showed the highest adherence to lipid-lowering therapy, with 99.1% of participants on treatment. The most commonly used statin was atorvastatin (74.1%), followed by rosuvastatin (23.1%). Combination or adjunctive therapies included ezetimibe (41.7%), while evolocumab (4.6%) and inclisiran (0.9%) were less frequently prescribed. Similarly, antiplatelet therapy was prescribed for 93.5% of patients—primarily aspirin (82.4%), with clopidogrel (47.2%) and ticagrelor (16.7%) used in combination or as alternatives. Beta-blocker therapy was prescribed for 88.9% of participants (see Table S1)—primarily bisoprolol (85.2%) and, less frequently, metoprolol (2.8%) and carvedilol (0.9%). Details about each medication category and sex differences are provided in Tables S3 and S4.

Regarding physical activity, 27.8% of all participants met the moderate-intensity recommendation, and <1% met the vigorous-intensity target, with no significant differences between patient groups (χ^2^ = 0.06, *p* = 0.804; χ^2^ = 3.03, *p* = 0.082, respectively). Only one participant from a non-tertiary hospital (3.7%) reported performing vigorous exercise (Figure 1, see Tables S1 and S5).

Nearly half of participants (46.3%) were non-smokers, 38.0% were past smokers, and 15.7% were current smokers. Smoking status showed no significant association with hospital type: χ^2^(2, *n* = 108) = 3.16, *p* = 0.206. Among patients with a history of smoking (*n* = 58), the majority (84.5%) reported smoking cigarettes only, while smaller proportions used sheesha (6.9%) or both forms (8.6%). Regarding smoking duration, 37.9% had smoked for over 30 years, 32.8% reported smoking for 16–30 years, and 29.3% had smoked for <15 years (see Tables S1 and S6).

Cardiac rehabilitation was achieved by 25% of patients. The referral rate did not differ between tertiary and non-tertiary groups: χ^2^(1, *n* = 108) = 1.33, *p* = 0.248. Adherence to non-pharmacological and clinical risk factor targets was nearly evenly distributed among the study participants. Approximately half of the sample (50.9%) did not meet the combined adherence criteria, while 49.1% achieved full adherence across the included lifestyle and clinical indicators. These adherence criteria included maintaining a normal BMI, meeting the recommended physical activity levels, achieving controlled LDL cholesterol, maintaining controlled blood pressure, achieving target fasting glucose levels, being referred to cardiac rehabilitation, and being a non-smoker (see Table S7). None of the participants achieved composite guideline adherence (Figure 1).

## 4. Discussion

To our knowledge, the 4S registry was the first to assess secondary prevention at follow-up among patients with prior acute myocardial infarction in Saudi Arabia. This pilot-phase study yielded several major findings. First, 20% of our patients had experienced multiple AMI events. Second, over half (53.7%) of the patients had uncontrolled blood pressure, and nearly 40% had uncontrolled glucose levels. Third, two-thirds (67%) of patients had uncontrolled LDL levels, over three-quarters were either overweight or obese (40.7% and 37%, respectively), and less than one-third (28.7%) met the recommended targets for weekly physical activity. Fourth, 33% of our participants were not receiving all four pillars of GDMT. Fifth, 15.7% were current smokers, and only 25% had been referred to cardiac rehabilitation. Sixth, none of the participants achieved the composite of guideline recommendations.

Patients with known cardiovascular disease (CVD) have a 20% increase in their absolute risk of experiencing another CVD event over 5 years, and this risk is highest within the first two years [1,2,17]. Among overall CVD events, nearly half are due to recurrent CVDs [1,2,17]. Our present findings revealed that nearly one-fifth of patients had already experienced multiple ACS events [1,2]. This implies that a large proportion of patients experience another event due to poor guideline adherence. A meta-analysis revealed that patients with established cardiovascular disease exhibited a 1.27-fold greater reduction in their absolute risk of major adverse cardiovascular events after 5 years of blood pressure lowering, compared with primary prevention patients [18]. However, despite blood pressure control, their residual risk remains 4.1-times higher than that of primary prevention patients [18]. Among our participants, less than half achieved the target blood pressure, with slightly better control among patients at non-tertiary hospitals (51.9%) compared to tertiary hospitals (44.4%). The International Action on Secondary Prevention through Intervention to Reduce Events (INTERASPIRE) study recruited 4548 patients from 14 different countries, and interviewed them at nearly a year after their atherosclerotic cardiovascular disease event [1]. The results showed that only 38.6% achieved a target blood pressure of <130/80 mmHg [1]. Similarly, the EUROASPIRE V study reported that 42% of their patients achieved a blood pressure of <140/90 mmHg [19]. The risk of death from cardiovascular diseases is increase up to six-fold in patients with type 2 diabetes mellitus [20]. The National Health and Nutrition Examination Survey (NHANES) demonstrated a 4.4-fold increase in the death rate secondary to diabetes alone among post-MI patients [20]. Within our present cohort, over one-third of patients had uncontrolled glucose levels, with higher proportions among patients at tertiary hospitals (44.1%). Consistently, the INTERASPIRE and EUROASPIRE V studies reported that 67% and 46%, respectively, of their patients showed abnormal glucose measurements and poor secondary prevention of diabetes (≥7.0%) [1].

The current estimate of the burden of LDL-C shows that it accounts for nearly 95 million disability-adjusted life years (DALYs), increasing drastically throughout the past 25 years [21]. Two-thirds of our study participants presented with LDL levels of ≥1.4 mmol/L, with higher proportions among patients at non-tertiary hospitals. In the DAUSSET study—which included 912 adult patients with established coronary artery disease (CAD) and treated for secondary prevention—only 41.7% were able to achieve the LDL-C goal of <70 mg/dL (1.8 mmol/L) [22]. Results from the multinational observational treatment of high- and very high-risk dyslipidemic patients for the prevention of cardiovascular events in Europe (SANTORINI) study and the EU-wide cross-sectional observational study of lipid-modifying therapy use in secondary and primary care (DA VINCI) study have revealed that nearly 20–30% of high-risk and very high-risk dyslipidemia patients achieved the 2019 ESC/EAS LDL guideline-recommended target [23,24,25]. This may be attributed to low usage of high-dose statin monotherapy and combination therapies [23,24,25]. Correspondingly, in the INTERASPIRE study, 83% of participants failed to control their LDL-C levels (<1.4 mmol/L) after one year from the index event [1]. According to the Joint European Societies (JES) guidelines on cardiovascular disease prevention, proper weight control is strongly recommended after a cardiovascular event to reduce the risk of diabetes, lipids, and elevated blood pressure [21]. Despite these recommendations, two-thirds (77.7%) of our patients were overweight or obese [26]. Data from the EUROASPIRE IV and V studies revealed that among 10,507 coronary heart disease patients, 16.4% showed a ≥5% increase in weight at 6–24 months after their event [26]. Patients with higher weights were also more likely to exhibit uncontrolled blood pressure and sugar levels [26]. Data extracted from population-based cohort studies including over 130 thousand participants with CVD demonstrated that increasing exercise volume has an inverse effect on mortality rates [27]. Every physical activity increase of 500 metabolic equivalent min per week (MET-min/wk) resulted in a 14% reduction in mortality in secondary prevention groups, while exercise-based rehabilitation provided an up to 63% reduction in mortality among CAD patients [27]. In alignment with the INTERASPIRE and EUROASPIRE studies, less than one-third of participants in our present study achieved the WHO weekly physical activity recommendations. The PURE study, which assessed over 200,000 patients, reported that less than half were physically active (performed more than 3000 MET-min/wk) [28]. Physical activity has been shown to reduce blood pressure, reduce serum lipids, increase lean body mass, and improve glucose control and kidney function [29]. The poor control of body weight and LDL, and physical inactivity, observed in our study may be attributed to suboptimal adherence to lifestyle recommendations, or inadequate guidance from healthcare practitioners.

The four pillars of secondary prevention are ACE/ARBs, lipid-lowering therapies, beta-blockers, and antiplatelet therapies [1]. Only two-thirds of our patients were taking all four pillars of GDMT, with the majority using anti-platelet therapy. The 2025 focused update of the 2019 ESC/EAS guidelines for the management of dyslipidemias and the 2025 ACC/AHA/ACEP/NAEMSP/SCAI Guideline for the Management of Patients with Acute Coronary Syndromes both recommend the upfront use of statins and ezetimibe in high-risk and very high-risk patients [30,31]. Nearly all of our patients were receiving lipid-lowering therapy (i.e., statins); however, less than half were being treated with combination therapy (i.e., with ezetimibe, evolocumab, or inclisiran). The limited use of these agents—despite their well-established efficacy—may have contributed to the high prevalence of uncontrolled LDL-C levels (67%). Beta-blockers were used by 88.9% of patients, and ACE inhibitors by 75.9%; however, over half of our patients had uncontrolled blood pressure. This may be attributed to low compliance or suboptimal dosing. A sub-cohort analysis of the PURE study in the Middle East (United Arab Emirates, Saudi Arabia, Occupied Palestinian Territory, and Islamic Republic of Iran) revealed that only 37.3% of the participants with prior coronary heart disease were using ≥3 of the pillars of GDMT [32]. Consistently, in the INTERASPIRE study, less than half of patients were prescribed all four treatments; antiplatelet and lipid-lowering therapies were most commonly used, followed by beta-blockers and ACE inhibitors. According to the WHO, smoking is the second leading cause of CVD, leading to nearly 10% of overall CVDs [33]. Recent reports from the US estimate that smoking costs >$300 billion each year [34]. A recent review of 68 studies, including over 80,000 participants, showed that smoking cessation for secondary prevention of CVDs resulted in decreased risks of non-fatal MI, non-fatal stroke, and all-cause mortality [35]. Our present data revealed that nearly half of our participants were either current or past smokers, with two-thirds of patients having smoked for at least 16 years. Smoking is associated with earlier CVD onset, by 5.1 years in males and by 3.8 years in females [34]. Referral to tobacco control clinics, or informing patients of the risk of smoking, could aid in smoking cessation [36].

Cardiac rehabilitation is performed with the aims of improving cardiovascular health and quality of life, lowering the risk of mortality, reducing physiological and psychological stress, and reducing recurrent hospitalizations [37]. The American Association of Cardiovascular and Pulmonary Rehabilitation, the Agency for Health Care Policy and Research, and American Heart Association have each emphasized the need for a comprehensive cardiac rehabilitation program, designed to optimize these aspects [37,38]. Cardiac rehabilitation remains underutilized globally. Our present study found that over three-quarters of patients were not referred to cardiac rehabilitation, consistent with findings from previous studies conducted in Saudi Arabia [39,40].

Secondary prevention plays a crucial role in limiting CVD recurrence; however, it has been a challenge to reach these recommended risk factor targets in most regions of the world. In our study, none of the patients were able to achieve the composite of guideline recommendations. The STARS-1 registry revealed that 20–40% of patients had uncontrolled CAD risk factors at the 1-year follow-up [5]. Pagidipati et al. investigated AMI patients with diabetes, focusing on secondary prevention parameters of cardiovascular disease—including aspirin use, lipid control, blood pressure control, use of angiotensin-converting enzyme inhibitors or angiotensin receptor blockers, and nonsmoking status—among 13,616 patients from 38 different countries [41]. They found that nearly two-thirds of patients with diabetes mellitus and cardiovascular disease did not achieve all five parameters [41]. Similarly, results from the INTERASPIRE study show that only 1% of the cohort achieved optimal guideline adherence [1,18]. These results highlight this problem on a global scale, and indicate the urgent need for optimal secondary prevention measures. Recent evidence from the World Heart Federation shows that the incidence of CVD could be reduced by 50% through the properly implemented use of polypills, as well as the use of a multidisciplinary approach to optimize all CVD risk factors [42].

The present study had several limitations. Although the study was designed to recruit consecutive patients, this was not verified, similar to in previous registries. Furthermore, this observational study was prone to inherent bias in the form of missing or incomplete information, and selection bias. Additionally, due to the small sample size of the current study, many of the results were not statistically significant; thus, these entities should be interpreted with caution. Future phases of the study will include a larger sample and will provide a comprehensive overview of adherence to guideline-recommended secondary prevention targets across Saudi Arabia, as well as a comparison of key outcomes across various demographics (e.g., tertiary vs. non-tertiary hospitals and male vs. female patients).

## Figures and Tables

**Table 1:** Socio-demographic and clinical characteristics of the study participants (*n* = 108).

Variable	Category	Frequency, n	Percentage, %
Age, years	Mean ± SD	58.4 ± 10.9	—
Gender	Male	87	80.6
	Female	21	19.4
Nationality	Saudi	83	76.9
	Non-Saudi	25	23.1
Type of care	Tertiary	81	75.0
	Non-tertiary	27	25.0
Time of inclusion, years	Mean ± SD	1.29 ± 0.51	
Hospitals	Tertiary Hospital 1, Riyadh	30	27.8
	Tertiary Hospital 2, Riyadh	26	24.1
	Tertiary Hospital 3, Riyadh	25	23.1
	Non-tertiary Hospital 1, Riyadh	15	13.9
	Non-tertiary Hospital 2, Qatif	12	11.1
Number of AMI events	One	86	79.6
	Multiple	22	20.4
Systolic blood pressure, mmHg	Mean ± SD	127.7 ± 17.8	—
Diastolic blood pressure, mmHg	Mean ± SD	73.9 ± 11.7	—
LDL-C, mmol/L	Mean ± SD	1.92 ± 0.92	—
Glucose level, HbA1c, %	Mean ± SD	7.09 ± 1.81	—
Height, cm	Mean ± SD	166.1 ± 7.2	—
Weight, kg	Mean ± SD	78.3 ± 15.8	—
BMI, kg/m2	Mean ± SD	28.3 ± 5.1	—
BMI categories	Underweight	2	1.9
	Normal weight	22	20.4
	Overweight	44	40.7
	Obese	40	37.0

AMI—acute myocardial infarction; BMI—body mass index; LDL-C—low-density lipoprotein cholesterol.

## Data Availability

All relevant data are included in the article.

## Supplementary Materials

The following supporting information can be downloaded at: https://www.mdpi.com/article/10.3390/jcdd13020100/s1, Table S1: Controlled Clinical Variables by Hospital Type; Table S2: Lp(a) Measurement (*n* = 108); Table S3: Prescription of Guideline-Directed Medical Therapy (*n* = 108); Table S4: Medication use by gender (*n* = 108); Table S5: Physical Activity Details; Table S6: Smoking Details; Table S7: Distribution of Total Adherence to Non-Pharmacological and Clinical Risk Factor Targets (*n* = 108).
