# Supplementary material for: Saudi Secondary Prevention Survey Study in Patients with Prior Acute Myocardial Infarction (4S Registry): Study Design and Pilot Phase Results
Source: J Cardiovasc Dev Dis. 2026 Feb 20;13(2):100. doi: 10.3390/jcdd13020100 (PMC12942633; doi:10.3390/jcdd13020100)
Supplement: Supplementary file 1 [file jcdd-13-00100-s001.zip › jcdd-4122354-supplementary.pdf]

Electronic Supplementary Material (ESM)

ESM 1

Table S1. Controlled Clinical Variables by Hospital Type

Variable	Tertiary	Non-tertiary	Total	χ²	df	p-value
**Blood Pressure Control**	**n = 81**	**n = 27**	**N = 108**	**0.4428**	**1**	**0.5058**
Controlled (<130/80 mmHg)	36 (44.4 %)	14 (51.9 %)	50 (46.3 %)			
Uncontrolled (≥130/80 mmHg)	45 (55.6 %)	13 (48.1 %)	58 (53.7 %)			
**Glucose Control**	**n = 68**	**n = 19**	**N = 87**	**3.2803**	**1**	**0.0701**
Controlled (HbA1c < 7%)	38 (55.9%)	15 (78.9%)	53 (60.9%)			
Uncontrolled (≥7%)	30 (44.1%)	4 (21.1%)	34 (39.1%)			
**Lipid Profile Control**	**n = 65**	**n = 23**	**N = 88**	**1.7526**	**1**	**0.1856**
Controlled (<1.4 mmol/L)	24 (36.9 %)	5 (21.7 %)	29 (33.0 %)			
Uncontrolled (≥1.4 mmol/L)	41 (63.1 %)	18 (78.3 %)	59 (67.0 %)			
**GDMT adherence (All four drugs)**	**n = 81**	**n = 27**	**N = 108**	**1.9815**	**1**	**0.1592**
Yes	51 (63.0%)	21 (77.8%)	72 (67.0%)			
No	30 (37.0%)	6 (22.2%)	36 (33%)			
**Anthropometric Indicators (BMI Categories)**	**n = 81**	**n = 27**	**N = 108**	**3.2727**	**1**	**0.3515**
Underweight	1 (1.2 %)	1 (3.7 %)	2 (1.9 %)			
Normal Weight	14 (17.3 %)	8 (29.6 %)	22 (20.4 %)			
Overweight	36 (44.4 %)	8 (29.6 %)	44 (40.7 %)			

Obese	30 (37.0 %)	10 (37.0 %)	40 (37.0 %)			
**Physical Activity**	**n = 81**	**n = 27**	**N = 108**			
150–300 min of moderate-intensity aerobic activity per week	23 (28.4%)	7 (25.9%)	30 (27.8%)	0.062	1	0.804
75–150 min of vigorous-intensity aerobic activity per week	0 (0%)	1 (3.7%)	1 (0.9%)	3.028	1	0.082
Doesn't perform activity	58 (71.6%)	19 (70.4%)	77 (71.3%)			
**Smoking habits (Smoking status)**	**n = 81**	**n = 27**	**N = 108**	3.156	2	0.206
Non-smoker	41 (50.6%)	9 (33.3%)	50 (46.3%)			
Past smoker	27 (33.3%)	14 (51.9%)	41 (38.0%)			
Current smoker	13 (16.0%)	4 (14.8%)	17 (15.7%)			
**Cardiac Rehabilitation Referral**	**n = 81**	**n = 27**	**N = 108**	**1.333**	**1**	**.248**
Referred	18 (22.2%)	9 (33.3%)	27 (25.0%)			
Not Referred	63 (77.8%)	18 (66.7%)	81 (75.0%)			
**Composite Guideline Adherence**	**n = 81**	**n = 27**	**N = 108**			
Achieved	0 (0%)	0 (0%)	0 (0%)	-----	-----	-----
Not achieved	81 (100%)	27 (100%)	108 (100%)			

ESM 2

Table S2. Lp(a) Measurement (N = 108)

Variable	Category	Frequency (n)	Percentage (%)
**Lipoprotein(a) measurement**	Yes	1	0.9
	No	107	99.1

ESM 3

Table S3. Prescription of Guideline-Directed Medical Therapy (N = 108)

Medication Type	Category	Frequency (n)	Percentage (%)
**ACE inhibitors/ARBs**	Yes	82	75.9
	No	26	24.1
**Types used (n = 80)**	Valsartan	26	32.5
	Perindopril	25	31.3
	Lisinopril	15	18.8
	Losartan	7	8.8
	Entresto	3	3.8
	Irbesartan	2	2.5
	Telmisartan	2	2.5
**Lipid-lowering therapy**	Yes	107	99.1
	No	1	0.9
**Types used**	Atorvastatin	80	74.1
	Rosuvastatin	25	23.1
	Ezetimibe	45	41.7
	Evolocumab	5	4.6
	Inclisiran	1	0.9
**Antiplatelet therapy**	Yes	101	93.5
	No	7	6.5
**Types used**	Aspirin	89	82.4
	Clopidogrel	51	47.2
	Ticagrelor	18	16.7
**Beta-blocker**	Yes	96	88.9
	No	12	11.1
**Types used**	Bisoprolol fumarate	92	85.2
	Metoprolol	3	2.8
	Carvedilol	1	0.9

ESM 4

No statistically significant gender differences were observed in the prescription of guideline-recommended cardioprotective GDMT. ACE inhibitor/ARB use was reported in 77.0% of males and 71.4% of females, $\chi^2(1, N = 108) = 0.29$, $p = .591$. Nearly all participants received lipid-lowering therapy (98.9% of males, 100% of females), $\chi^2(1, N = 108) = 0.24$, $p = .622$.

Antiplatelet therapy was used by 95.4% of males and 85.7% of females, showing a nonsignificant trend toward higher use in men, $\chi^2(1, N = 108) = 2.62$, $p = .106$. Similarly, β-blocker use was high and comparable between males (88.5%) and females (90.5%), $\chi^2(1, N = 108) = 0.07$, $p = .796$. Lastly, concurrent use of all four cardioprotective drugs was slightly more common among males (69.0%) than females (57.1%), $\chi^2(1, N = 108) = 1.06$, $p = .302$.

Table S4. Medication use by gender (N = 108)

Medication / Indicator	Male (n=87)	Female (n=21)	Pearson χ^2	df	p-value
**ACEi/ARB (Yes)**	**67 (77.0%)**	**15 (71.4%)**	0.288	1	.591
- No	20 (23.0%)	6 (28.6%)			
**Lipid-lowering (Yes)**	**86 (98.9%)**	**21 (100.0%)**	0.244	1	.622
- No	1 (1.1%)	0 (0.0%)			
**Antiplatelet (Yes)**	**83 (95.4%)**	**18 (85.7%)**	2.619	1	.106
- No	4 (4.6%)	3 (14.3%)			
**β-blocker (Yes)**	**77 (88.5%)**	**19 (90.5%)**	0.067	1	.796
- No	10 (11.5%)	2 (9.5%)			
**All four drugs (Yes)**	**60 (69.0%)**	**12 (57.1%)**	1.064	1	.302
- Otherwise	27 (31.0%)	9 (42.9%)			

ESM 5

Table S5. Physical Activity Details

Variable	Category	Frequency (n)	Percentage (%)
**Moderate physical activity (150–300 min/week)**	Yes	30	27.8
	No	78	72.2
**Vigorous physical activity (75–150 min/week)**	Yes	1	0.9

	No	107	99.1
**Type of physical activity**	None	77	71.3
	Endurance	30	27.8
	Strength	1	0.9

ESM 6

Table S6. Smoking Details

Variable	Category	Frequency (n)	Percentage (%)
**Smoking status**	Non-smoker	50	46.3
	Past smoker	41	38.0
	Current smoker	17	15.7
**Type of smoking (n = 58)**	Cigarettes only	49	84.5
	Sheesha	4	6.9
	Both	5	8.6
**Years of smoking (n = 58)**	<15 years	17	29.3
	16–30 years	19	32.8
	>30 years	22	37.9

ESM 7

Table S7. Distribution of Total Adherence to Non-Pharmacological and Clinical Risk Factor Targets (N = 108)

Total Adherence (No Drug Component)	Frequency (n)	Percent (%)
Not adherent	55	50.9
Adherent	53	49.1
**Total**	**108**	**100.0**

Note. Total adherence (no drug component) was defined as meeting all seven of the following criteria: normal BMI category, adherence to recommended physical activity levels, controlled LDL cholesterol (<1.4 mmol/L), controlled blood pressure (<130/80 mmHg), controlled fasting glucose (<7%), a Referred to Cardiac rehabilitation, and non-smoking status.

ESM 8

As shown in Table A, only a small proportion of participants (11.1%) achieved composite adherence—meeting all targets for pharmacological therapy and clinical control—while the majority (88.9%) were non-adherent.

Table A. Distribution of Composite Adherence Among the Study Participants (N = 108)

Composite Adherence	Frequency (n)	Percent (%)
Adherent	12	11.1
Non-adherent	96	88.9
**Total**	**108**	**100.0**

Note. Composite adherence was defined as meeting all of the following criteria: use of all four evidence-based drug classes (antiplatelet, β-blocker, ACE inhibitor/ARB, and statin), controlled blood pressure (<130/80 mmHg), controlled fasting glucose (<7%), and controlled LDL cholesterol (<1.4 mmol/L).

A Chi-square test indicated a statistically significant association between composite adherence and type of care, $\chi^2(1, N = 108) = 4.50$, $p = .034$. Patients in non-tertiary hospitals demonstrated higher adherence (22.2%) than those in tertiary care (7.4%) (Table B).

Table B. Association Between Composite Adherence 1 and Type of Care (N = 108)

Composite Adherence 1	Tertiary Caren (%)	Non-tertiary Caren (%)	Totaln (%)	χ^2 (df = 1)	p
Adherent	6 (7.4%)	6 (22.2%)	12 (11.1%)	4.50	.034
Not adherent	75 (92.6%)	21 (77.8%)	96 (88.9%)	—	—
**Total**	**81 (100%)**	**27 (100%)**	**108 (100%)**		

ESM 9

As presented in Table A, 22.2% of participants achieved Composite Adherence 2, indicating that they met targets for pharmacological therapy, blood pressure, and glucose control simultaneously. In contrast, the majority (77.8%) did not meet all these criteria.

Distribution of Composite Adherence Among the Study Participants (N = 108)

Composite Adherence 2	Frequency (n)	Percent (%)
Adherent	24	22.2
Not adherent	84	77.8
**Total**	**108**	**100.0**

Note. Composite Adherence 2 was defined as meeting all of the following criteria: use of all four evidence-based drug classes (antiplatelet, β-blocker, ACE inhibitor/ARB, and statin), controlled blood pressure (< 130/80 mmHg), and controlled fasting glucose (< 7%).

A chi-square test showed no significant association between composite adherence and type of care, $\chi^2(1, N = 108) = 2.57$, $p = .109$. Adherence was higher among non-tertiary patients (33.3 %) than tertiary patients (18.5 %), though the difference did not reach statistical significance (Table B).

Table B. Association Between Composite Adherence 2 and Type of Care (N = 108)

**Composite Adherence 2**	**Tertiary Caren (%)**	**Non-tertiary Caren (%)**	**Totaln (%)**	**χ^2 (df = 1)**	**p**
Adherent	15 (18.5 %)	9 (33.3 %)	24 (22.2 %)	2.57	.109
Not adherent	66 (81.5 %)	18 (66.7 %)	84 (77.8 %)	—	—
**Total**	**81 (100 %)**	**27 (100 %)**	**108 (100 %)**		
